# Successful Salvage of a Renal Allograft after Acute Renal Vein Thrombosis due to May-Thurner Syndrome

**DOI:** 10.1155/2012/390980

**Published:** 2012-10-22

**Authors:** Omkar U. Vaidya, Todd Buersmeyer, Rebecca Rojas, Bart Dolmatch

**Affiliations:** ^1^Division of Nephrology, Department of Internal Medicine, UT Southwestern Medical Center, 5323 Harry Hines Boulevard, Dallas, TX 75390-8856, USA; ^2^Department of Intervention Radiology, UT Southwestern Medical Center, 5323 Harry Hines Boulevard, Dallas, TX 75390, USA

## Abstract

A 68-year-old Caucasian female with a past medical history of a deceased donor kidney transplant four months prior was admitted with a two-day history of anuria and acute kidney injury. A renal ultrasound demonstrated thrombus in the transplanted kidney's renal vein that extended into the left iliac vein as well as into the left femoral venous system. Catheter-guided tissue thrombolytics were infused directly into the clot. Within twelve hours of initiating thrombolytic infusion, there was brisk urine output. Interval venography demonstrated decreasing clot burden. At the time of discharge her creatinine was 0.78 mg/dL, similar to her baseline value prior to presentation. The patient was noted to have May-Thurner syndrome on intravascular ultrasound (IVUS). Angioplasty followed by stent placement was done. Unique to our case report was the timing of the presentation of renal vein thrombosis (four months after transplant) and the predisposing anatomy consistent with May-Thurner syndrome, which was diagnosed with IVUS and successfully treated with local thrombolytics.

## 1. Case Presentation

Renal vein thrombosis secondary to May-Thurner syndrome, postrenal transplantation, is not common. Early diagnosis and intervention are important in preventing renal allograft loss. 

A 68-year-old nonobese Caucasian female recipient of a left lower quadrant deceased donor kidney transplant due to adult polycystic kidney disease presented four months after transplantation with a two-day history of anuria. Serum creatinine at presentation was 2.20 mg/dL (baseline 0.8 mg/dL). Her immunosuppressant regimen included tacrolimus and prednisone. Renal Doppler ultrasound demonstrated allograft renal vein thrombosis extending into the left iliac and femoral venous systems. Initially, open surgical exploration and thrombectomy of the clotted vein was planned and preoperatively an inferior vena caval filter was placed. However, the patient had minimal urine output since admission and the ultrasound also demonstrated retained transplant arterial flow. Thinking of it as less invasive and having a possibility of success, catheter-based thrombolysis was attempted. 

**Figure 1:**
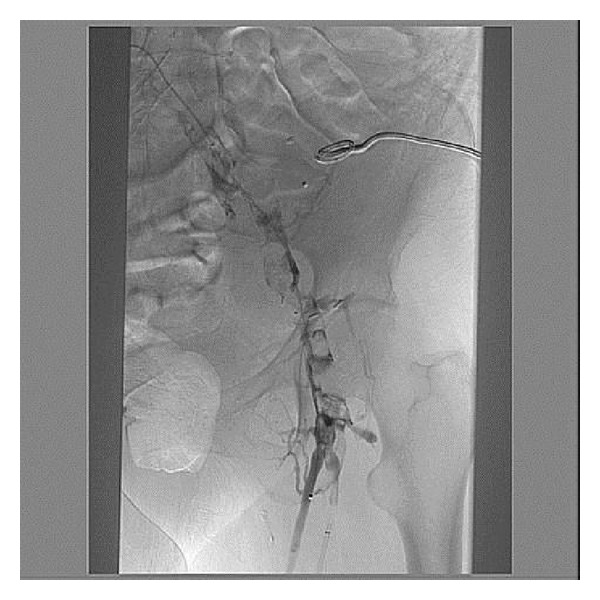
Initial diagnostic venography at presentation prior to thrombolysis; left iliac vein.

**Figure 2:**
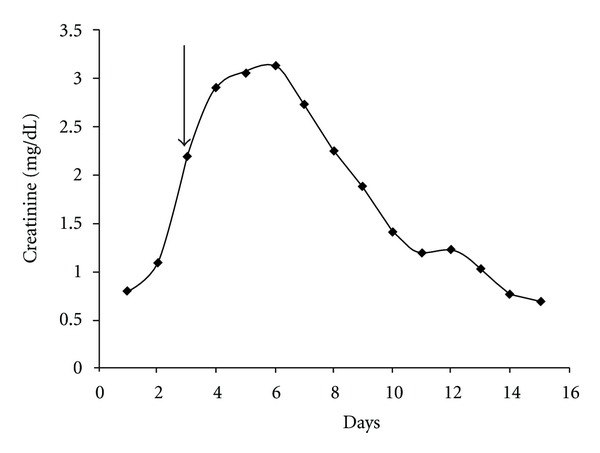
The *x*-axis is time (days) and the *y*-axis serum creatinine values in mg/dL. Elevation of serum creatinine was seen at the time of admission (arrow) which peaked and then gradually returned to baseline after successful thrombolysis, angioplasty, and stent placement.

Two infusion catheters were placed under fluoroscopic guidance: one to treat the transplant and juxta-anastomotic iliac veins and the other for the iliofemoral venous system distal to the allograft (done within twelve hours after presentation). (Figure 1). Tissue plasminogen activator (tPA) was infused directly into the clot with a total dose of 1 mg/hr (patient weight = 83 kg) divided evenly between two catheters. Heparin was also started at 500 Units/hour. Afterwards, increasing urine output was seen within 12 hours. Serum creatinine peaked at 3.14 mg/dL and then gradually returned to baseline after successful thrombolysis, angioplasty, and stent placement (Figure 2). tPA was continued for approximately 2 days.

**Figure 3:**
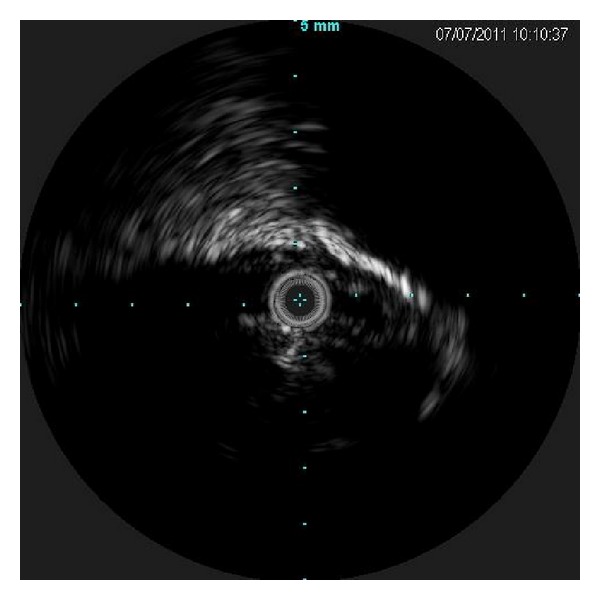
Intravascular ultrasound (IVUS) in the left iliac vein (anterior at top of image) demonstrating compression from the adjacent right iliac artery (2 o'clock position) before stenting.

**Figure 4:**
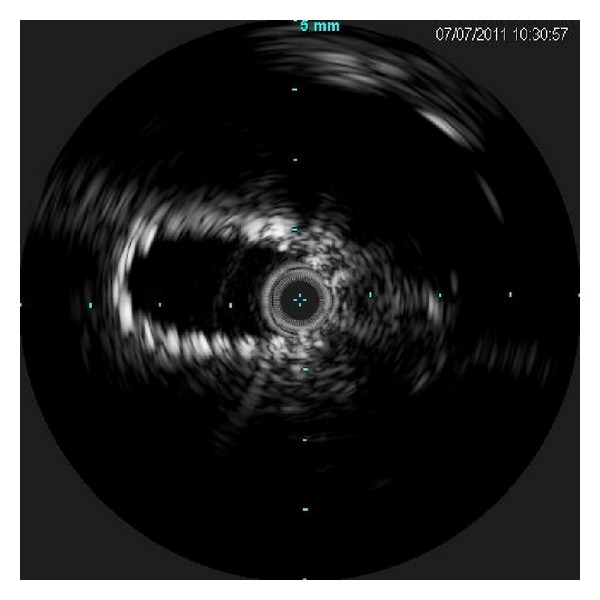
Poststent placement intravascular ultrasound (IVUS) in the left iliac improvement in lumen size.

Intraprocedural intravascular ultrasound (IVUS) of left iliac vein demonstrated compression of left iliac vein between the right iliac artery and vertebra (Figure 3). Given this, angioplasty followed by stent placement (self-expanding nitinol 12 mm Smart stent, Cordis Corporation, Miami, FL, USA) was performed. Poststent placement IVUS demonstrated improvement in lumen size (Figure 4). Warfarin was started on day 4 and heparin bridge was continued till day 8, when the INR became therapeutic (2.5–3.5). The native kidneys, though large from polycystic disease, did not contribute to the vascular compression.

**Figure 5:**
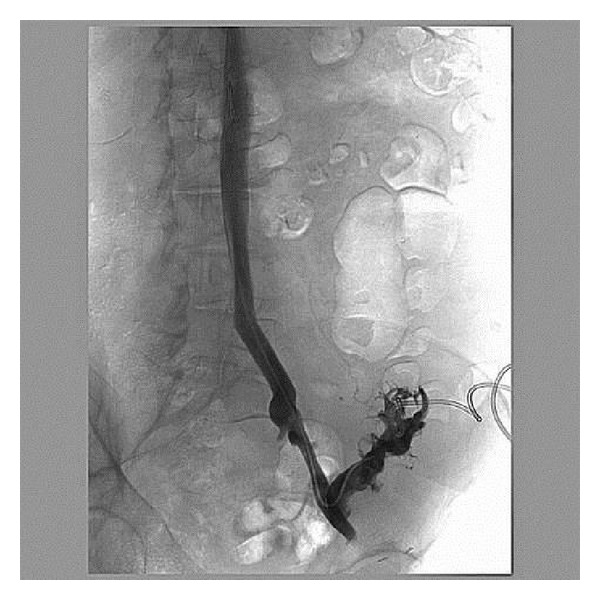
Follow-up venogram demonstrating widely patent iliac vein, stent, and transplant allograft vein.

At the time of discharge creatinine was 0.78 mg/dL, similar to baseline. Followup venography at discharge and 3 months later demonstrated complete stent patency without residual thrombus (Figure 5). After 4 months, serum creatinine was 0.71 mg/dL and Warfarin was stopped. Creatinine nearly 1 year after procedure remained similar at 0.8 mg/dL. 

## 2. Discussion

Renal vein thrombosis is an important but uncommon cause of allograft failure with an incidence of 1% [1]. Generally, it occurs in the early postoperative period, typically one to 2 weeks following surgery [2]. Risk factors associated with renal vein thrombosis include membranous nephropathy, systemic lupus erythematosis, antiphospholipid antibody syndrome, oral contraception, and hereditary thrombophilia secondary to protein C or factor V deficiency [3–7] amongst others. Catheter-directed thrombolysis followed by stent placement has been shown to be a successful treatment for renal vein thrombosis [7]. 

May-Thurner syndrome, originally described by May and Thurner in 1956, is an anatomic variant where the left common iliac vein is narrowed due to the compression between the overlying right common iliac artery and the underlying lumbar vertebrae [8]. May-Thurner syndrome has been described in renal transplant patients [9–11]. 

Our patient was noted to have renal vein thrombosis in the late posttransplant period secondary to underlying May-Thurner syndrome which was diagnosed with the help of IVUS. Moreover, our case is unique in showing successful catheter-directed treatment of the renal allograft and systemic venous thrombolysis in combination with endovascular stent placement, restoring the patient's allograft function as a treatment modality. Unique to our case was also the sustained success of our intervention at one-year followup.
